# Screening and Characterisation of Antimicrobial Properties of Semisynthetic Betulin Derivatives

**DOI:** 10.1371/journal.pone.0102696

**Published:** 2014-07-17

**Authors:** Shafiul Haque, Dorota A. Nawrot, Sami Alakurtti, Leo Ghemtio, Jari Yli-Kauhaluoma, Päivi Tammela

**Affiliations:** 1 Centre for Drug Research (CDR), Faculty of Pharmacy, University of Helsinki, Helsinki, Finland; 2 Division of Pharmaceutical Chemistry, Faculty of Pharmacy, University of Helsinki, Helsinki, Finland; 3 VTT Technical Research Centre of Finland, Espoo, Finland; University at Buffalo, SUNY, United States of America

## Abstract

Betulin (lup-20(29)-ene-3*β*, 28-diol) is a naturally occurring triterpene, which is found in substantial amounts from the outer bark of birch trees. A library of 51 structurally diverse semisynthetic betulin derivatives was screened against five bacterial strains, *Enterobacter aerogenes*, *Escherichia coli*, *Enterococcus faecalis*, *Pseudomonas aeruginosa, Staphylococcus aureus* and a fungal strain *Candida albicans*, using broth microdilution assays. Primary antimicrobial screening at 50 µM concentration led to the identification of five compounds showing antimicrobial properties (inhibition of growth by >70% against one or more microbial strains). According to the dose-response results, 28-*O*-(*N*-acetylanthraniloyl)betulin (compound **5**) was the most active, showing MIC_90_ of 6.25 µM against two Gram-positive bacteria, *E. faecalis* and *S. aureus*. However, the activity of this compound was affected by albumin binding, which was demonstrated by the loss of activity in a host-pathogen co-culture assay as well as in the antibacterial assay in the presence of increased concentration of albumin. Furthermore, the effects on mammalian cells were evaluated by cytotoxicity assessment on hepatocyte cell culture after 24 h exposure to the compounds. Betulinic aldehyde (**18**), betulin-28-oxime (**31**) and hetero cycloadduct with acetoxy groups at carbon atoms 3 and 28 and ethyl substituent at the triazolo ring (**43**) displayed cytotoxicity towards hepatocytes, with IC_50_ values of 47, 25 and 16 µM, respectively. The IC_50_ value for 28-*O*-(*N*-acetylanthraniloyl)betulin (**5**) was 56 µM. The current study presents an insight into using betulin scaffold for developing derivatives with antibacterial potential, and furthermore the necessity of in-depth analysis of found actives through selectivity profiling and follow-up studies including *in silico* ADMET predictions.

## Introduction

According to the World Health Organisation (WHO), infectious and parasitic diseases account for two to five of the top ten causes of deaths in the world. The need for new antimicrobials has been recognised by the WHO, the European Centre for Disease Control and Prevention, as well as by the European Medicines Agency. In 2009, the WHO declared antibiotic resistance as one of the three foremost threats to public health. Therefore, the theme of the World Health Day, 2011 was “antimicrobial resistance: no action today and no cure tomorrow” [1]–[3]. Bacterial infections alone are the cause of around two million deaths globally and it is estimated that bacterial pathogens probably infect more than one-third of the population around the world [4].

**Figure 1:**
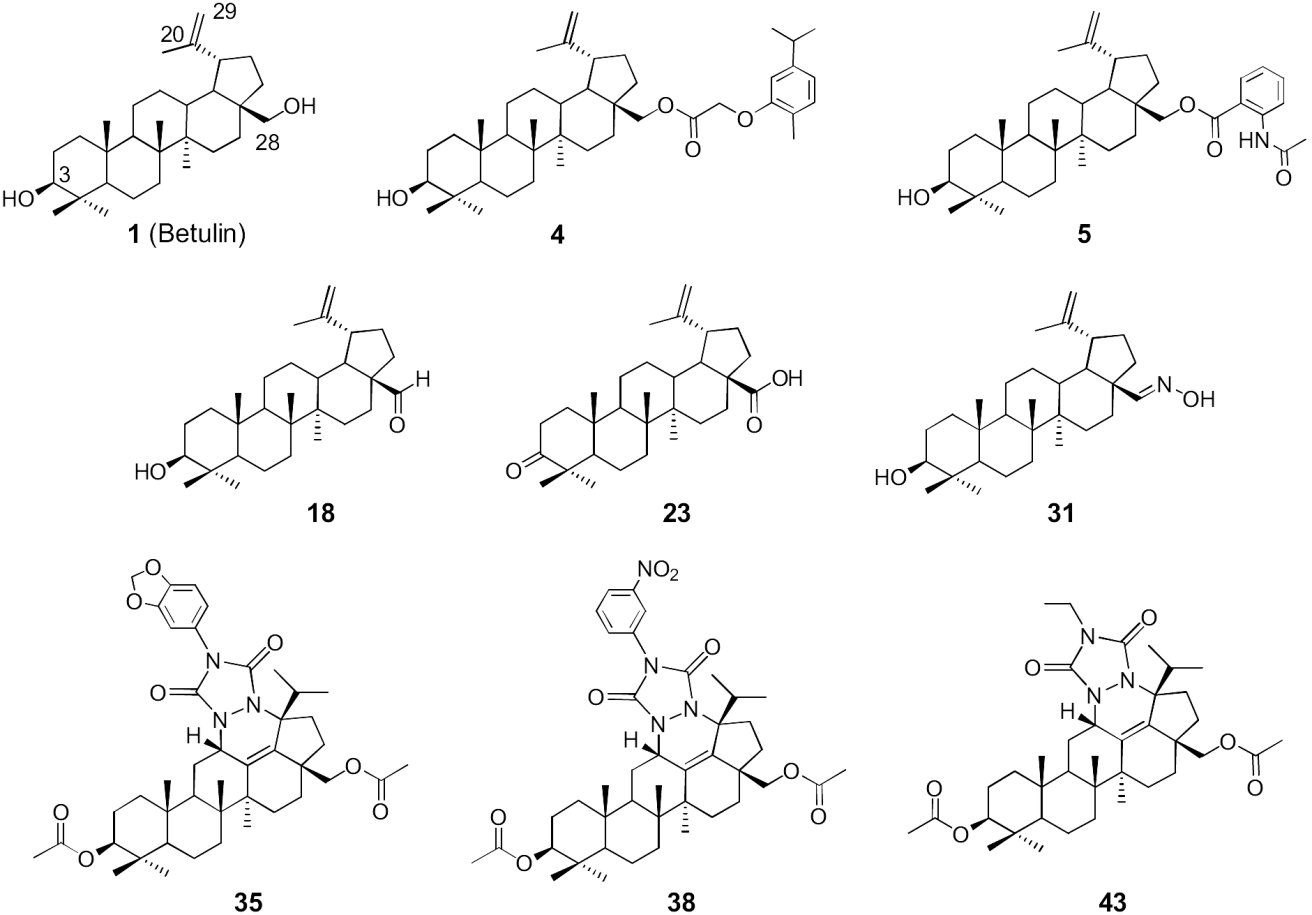
Chemical structures of betulin and the most active betulin derivatives. Betulin (**1**), betulinyl 28-carboxymethoxycarvacrolate (**4**), 28-*O*-(*N*-acetylanthraniloyl)betulin (**5**), betulinic aldehyde (**18**), betulonic acid (**23**), betulin-28-oxime (**31**), and heterocyclic derivatives with 1,3-dioxol-5-yl (**35**), 3-nitrophenyl (**38**) and ethyl (**43**) attached to the nitrogen atom in the triazolo ring.

Novel medicinal lead compounds are sought by assaying large compound collections, which can be retrieved from natural sources or produced by synthetic chemistry. For decades the majority of drugs have been discovered from natural products: 34% of new small-molecules introduced as drugs worldwide during 1981–2010 can be traced to, or were inspired by, natural products [5]. Natural products are recognised as good sources of scaffolds, which can be employed for generating focused, semisynthetic libraries. One potential scaffold is betulin (lup-20(29)-ene-3*β*, 28-diol), (Fig. 1, compound **1**), a pentacyclic triterpenoid possessing a lupane skeleton. The main source of betulin are birch trees (*Betula* sp., Betulaceae), which are widespread in the northern latitudes of the world. Noteworthy, there is presently no considerable use of this readily isolable compound. The betulin content of the outer part of bark is ranging up to 35%, depending on the birch species, site of ground, conditions and the age of a tree [6]. Moreover, it has been estimated that pulp mill producing 200000 t/a of birch kraft pulp could theoretically produce 3000 t/a of betulin [7].

Birch trees have been widely used as folk remedies, in particular for their wound healing properties [8]. The birch bark oil is also used to treat the skin diseases i.e. eczema and psoriasis [9]. Moreover, the white birch bark has been used by Native Americans to prepare teas and other beverages to treat digestive tract infections [10].

Betulin, the main constituent of birch trees, has been investigated previously for its various biological properties, and has been found to possess a broad range of activities. Amongst those are antiseptic, anti-inflammatory, antiviral properties, as well as antibacterial, antifungal and antitumour activities [8], [11], [12]. Antimicrobial activity of betulin and its derivatives have been reported against *Streptococcus pyogenes* with a minimum inhibitory concentration (MIC) of 85 µg/mL, and considerable activity has also been observed against other bacteria, such as *Escherichia coli, Staphylococcus aureus*, and *Enterococcus faecalis* [11], [13]. They have been also found to possess significant antifungal activities against two *Candida* species, *Candida albicans* and *Candida krusei* [12]. One of the most interesting derivative, betulinic acid (compound **19**, Table S1) has been previously reported for its antimalarial, anti-inflammatory, antioxidant, antiprotozoal, anthelmintic, and antifungal activities [14]–[16]. It has also been considered for its anti-HIV activity, as well as for specific cytotoxicity against various tumour cell lines [17], [18]. However, it has been found to possess only mild antibacterial activity against *Bacillus subtilis* and *E. coli* [19].

This study presents the antimicrobial properties of semisynthetic betulin derivatives against six different microbial strains, supported by a series of selectivity and follow-up studies, in order to evaluate the true potential of the most active derivatives as leads for antibacterial drug development. These studies included *in silico* prediction of ADMET properties to assess and prioritise the most promising compounds for further hit to lead optimisation process.

## Materials and Methods

### Betulin derivative library

The chemical synthesis and characterisation of the 51 betulin derivatives (chemical structures are presented in the Tables S1 and S2) screened in this study have been previously reported [20], [21]. 10 mM stock solutions of tested samples were prepared in 100% dimethyl sulphoxide (DMSO, Merck) and stored at −20°C. For the assays, stock solutions were diluted with the assay media. The final DMSO concentration in the experiments was 0.5–2.0%, and did not have any effect on the growth of the tested strains.

### Reference compounds

Clinically used antimicrobial agents were selected based on international guidelines for antimicrobial testing and were utilised as reference compounds in the antimicrobial assays. The compounds were purchased from Sigma, Fluka and ICN Biomedicals. Stock solutions of the reference compounds were prepared either in double distilled sterile MilliQ water or in DMSO. Water solutions were sterilised through a 0.22 µm filter (VWR International). Before the primary antimicrobial screening, dose-response experiments were carried out in order to determine the MIC_90_ of the reference compounds towards their target strains, and to validate the assay performance (MIC_90_ values are presented in Table 1). During the primary screening, these reference compounds were used as positive controls at the determined MIC on every plate.

**Table 1:** Antimicrobial and cytotoxic effects of the most active betulin derivatives at 50 µM concentration (thresholds: antimicrobial activity >70%, cytotoxicity >50%).

	Inhibitory effect (%)						Cytotoxicity (%)
*No*	*E. aerogenes*	*E. coli*	*E. faecalis*	*P. aeruginosa*	*S. aureus*	*C. albicans*	*Huh-7 cells*
	*ATCC 13048*	*ATCC 25922*	*ATCC 29212*	*ATCC 27853*	*ATCC 25923*	*ATCC 90028*	
**4**	9.9±3.3	–1.2±1.2	**72.3**±**4.0**	3.0±3.0	–17.2±0.7	31.4±9.1	–4.6±3.6
**5**	25.2±2.0	0.5±3.1	**98.5**±**0.2**	12.3±2.3	**100.2**±**0.1**	10.0±5.2	30.0±3.0
**18**	13.5±2.6	2.7±2.4	39.6±8.0	3.9±0.6	–35.5±3.2	6.9±8.1	**61.3**±**9.5**
**23**	21.8±4.1	9.1±8.9	**74.3**±**0.8**	9.5±2.5	51.1±7.3	25.2±11.0	17.8±12.6
**31**	14.6±0.9	1.9±4.0	41.0±3.4	5.4±2.2	6.4±3.4	–73.5±43.9	**61.5**±**2.0**
**35**	14.2±1.6	3.4±3.1	**70.0**±**0.9**	5.5±1.0	5.9±1.2	4.4±12.9	–0.1±3.6
**38**	19.3±3.0	8.8±2.6	**73.9**±**1.4**	7.6±1.4	6.7±1.5	–13.8±13.3	8.3±3.9
**43**	18.5±2.2	–3.7±4.3	57.1±4.4	1.7±1.7	2.9±0.7	–9.7±14.1	**86.0**±**2.0**
Positivecontrol	Gentamycin	Gentamycin	Ciprofloxacin	Gentamycin	Ciprofloxacin	Amphotericin B	Polymyxin B sulphate
(MIC_90_)	(0.5 µg/mL)	(2 µg/mL)	(1 µg/mL)	(8 µg/mL)	(0.5 µg/mL)	(2 µg/mL)	(15 000 IU/mL)

Values represent the mean ± SD of 3–6 replicates. Inhibitory effects of the most active samples are in bold. Primary screening results for all tested compounds are available in Tables S1 and S2.

### Microbial strains and culture conditions

Gram-negative strains *Escherichia coli* (ATCC 25922), *Enterobacter aerogenes* (ATCC 13048) and *Pseudomonas aeruginosa* (ATCC 27853), Gram-positive strains *Staphylococcus aureus* (ATCC 25923) and *Enterococcus faecalis* (ATCC 29212), and fungal strain *Candida albicans* (ATCC 90028) were obtained from Microbiologics Inc., and used for the antimicrobial screening. Strains were selected according to the guidelines set for clinical laboratories by Clinical and Laboratory Standards Institute [CLSI, formerly National Committee on Clinical Laboratory Standards [22]] and the European Committee on Antimicrobial Susceptibility Testing (EUCAST). Bacterial strains were grown on Mueller Hinton II Agar (MHA) (BBL, BD) and Mueller Hinton II broth (MHB) (BBL, BD), whereas *Candida* was initiated on Sabouraud Dextrose Agar (SDA) (Difco, BD) plates. Media were prepared into MilliQ water according to manufacturer’s instruction and autoclaved at 121°C for 15 min. Bacteria were plated on MHA plates and incubated at 37°C for 16–18 h. Bacterial suspension for the assays was prepared by subculturing the bacteria into MHB and by incubating at 37°C for 16–20 h at 110 rpm prior to the assay. *Candida* strain was grown on SDA plates at 27°C for 16–18 h and suspended into sterile 0.9% saline for the assay.

### Mammalian cell lines and culture conditions

Huh-7 cells (derived from human hepatocellular carcinoma) were obtained as a gift from Prof. Ralf Bartenschlager (University of Heidelberg, Germany) and used for assessing the cytotoxicity against mammalian cells. HL cells (a heteroploid cell line used for propagating respiratory viruses) were used as a host cell line in the host-pathogen co-culture assay. Huh-7 cells were maintained in Dulbecco’s Modified Eagle Medium (DMEM) supplemented with 10% fetal bovine serum (FBS, Gibco), 100 µM non-essential amino acids, 2 mM L-glutamine and 100 IU/mL of penicillin and 100 µg/mL of streptomycin. HL cells were maintained in RPMI-1640 medium supplemented with 7.5% of FBS (Lonza), 2 mM L-glutamine, and 100 IU/mL of penicillin and 100 µg/mL streptomycin. Both cell lines were cultured at 37°C, 5% CO_2_ and 95% humidity.

### Microdilution assay to assess the antimicrobial activity

Antimicrobial screening assays were performed by broth microdilution method following the guidelines of CLSI and EUCAST. Bacterial suspensions were prepared as mentioned earlier and diluted with MHB to obtain a final inoculum of 5×10^5^ colony-forming units (cfu)/mL in the assay for all the bacteria (based on OD_620_ values previously calibrated against plate counts). *Candida* suspension was prepared into sterile 0.9% saline solution as stated earlier. The suspension was adjusted to yield final inoculum of 2.5×10^3^ cfu/mL by diluting into RPMI-1640 media (with L-glutamine, w/o NaHCO_3_ and supplemented with 2% glucose and 0.165 M MOPS, pH 7; Lonza). Assays were carried out in clear 96-well microtitre plates and initiated by dispensing an equal volume of microbial suspension and sample solution diluted into the assay medium into the wells. The plates were incubated for 24 h at 37°C (for *Candida* the incubation was at 35°C for 48 h) with agitation. Absorbance was measured at 620 nm with Victor plate reader (Wallac 1420, Perkin Elmer) at 0, 4 and 24 h with the bacteria, and at 0, 24 and 48 h with *Candida*. The antimicrobial activity of the samples was calculated from the absorbance values by comparing to the controls, and expressed as a percentage inhibition of growth. All the betulin derivatives were first assayed at the single concentration of 50 µM (two separate experiments, each with three replicates). Compounds showing inhibitory activity >70% in the primary screen were selected for dose-response experiments to determine the minimum inhibitory concentration (MIC_90_) (with two-fold dilution using nine concentrations between 100 and 0.39 µM). The MIC_90_ value was expressed as the lowest concentration of compound that inhibited the microbial growth by >90%. The MIC_90_ results are means of three replicates from two separate experiments.

The effect of bovine serum albumin (BSA) on antimicrobial activity was assessed by using the *S. aureus* microdilution assay in a similar manner as described above, but with increasing concentrations of BSA, ranging from 0.023 nM to 25 µM [23]. The concentration of the betulin derivative, compound **5**, was 6.25 µM (the MIC_90_ against *S. aureus*).

### Host-pathogen co-culture assay

Betulin derivatives with promising antimicrobial activity in the primary screening were assayed with host-pathogen co-culture assay previously described by Kleymann & Werling [24]. Briefly, HL cells used as the host cell were seeded at 40000 cells/well on black-walled microplates (Perkin Elmer) and incubated at 37°C, 5% CO_2_ and 95% humidity for 24 h. Cell monolayers were infected with 2000 cfus of *S. aureus* in RPMI 1640 media without antibiotics. Compounds were added to the media at different concentrations selected on the basis of the MIC determinations. Ciprofloxacin was used as a positive control. Co-cultures were incubated for 3 days at 37°C, 5% CO_2_ and 95% humidity. Following the incubation period the cells were washed with 200 µL 6.7 mM PBS, pH 7.4, and finally, 200 µL 6.7 mM PBS, pH 7.4 containing 10 µg/mL fluorescein diacetate (FDA) was added to the cells to evaluate the survival of the host cells. After 45 min incubation at room temperature (RT), fluorescence was measured at 485 nm excitation and 538 nm emission with a Victor plate reader.

### Cytotoxicity against mammalian cells

The effect of the betulin derivatives on metabolic activity of Huh-7 cells was evaluated by measuring the intracellular ATP content of the cells after 24 h exposure to the compounds. In brief, cells were seeded at 20000 cells/well on white-walled microplates (PerkinElmer) and incubated at 37°C, 5% CO_2_ and 95% humidity. After culturing the cells overnight, the medium was replaced with the assay media (5% FBS) containing the sample and the cells were incubated for 24 h. Polymyxin B sulphate (15000 IU/mL) was used as a positive control. Following the incubation period, Promega’s CellTiter-Glo Cell Viability Assay was used to determine the intracellular ATP levels according to the manufacturer’s instructions. Cells were washed with 100 µL 6.7 mM PBS, pH 7.4 and 50 µL of fresh assay media and 50 µL of the CellTiter-Glo reagent were added into the wells. After 2 min shaking, followed by 10 min incubation at RT, luminometric signal was measured using Varioskan Flash plate reader (Thermo Fisher Scientific).

Compounds were initially tested at single concentration of 50 µM (*n* = 3), and based on these results, the most potent compounds were subjected to the determination of IC_50_ values. The IC_50_ values were calculated by fitting the data from the dose-response experiments (concentration range between 100 µM and 1.56 µM was selected according to the potency of the compounds) into sigmoidal dose-response curves by using the Origin software (OriginLab Corp.).

### *In silico* prediction of ADMET properties and toxicity end-points

All computational studies were carried out with Discovery Studio version 3.5 (DS) from Accelrys Discovery Studio (Accelrys Inc.) using Hewlett Packard computer system (Linux Pentium) with 2 CPUs. ADMET descriptors protocol in DS uses the quantitative structure relationship (QSAR) to estimate the human intestinal absorption, aqueous solubility, blood brain barrier, cytochrome P450 2D6, hepatotoxicity and plasma protein binding. Human Intestinal Absorption (HIA) is predicted after oral administration [25], [26]. Intestinal absorption is defined as a percentage absorbed rather than as a ratio of concentrations. A well-absorbed compound is one that is absorbed at least 90% into the bloodstream in humans. Aqueous solubility model uses linear regression to predict the solubility of each compound in water at 25°C [27]. Blood Brain Barrier model predicts with a quantitative linear regression, the blood-brain penetration (blood brain barrier, BBB) after oral administration [25]. The cytochrome P450 2D6 (CYP2D6) model predicts CYP2D6 enzyme inhibition [28]. CYP2D6 is involved in the metabolism of a wide range of substrates in the liver and its inhibition by a drug constitutes majority of the cases of drug-drug interaction. The hepatotoxicity model predicts potential organ toxicity from a wide range of available literature data of compounds and substances known to exhibit liver toxicity [29]. The plasma protein-binding model predicts whether a compound will be highly bound (≥90% bound) to carrier proteins in the blood [30]. TOPKAT (Toxicity Prediction Komputer Assisted Technology) support assessment with quantitative structure-toxicity relationship (QSTR) models of specific toxicological end-points. These include aerobic biodegradability, mutagenicity (Ames test), developmental toxicity potential, rodent carcinogenicity, ocular irritation, skin sensitisation and rat oral LD50. Aerobic biodegradability model computes the probability of a query structure to be capable of aerobic biodegradation [31]. The mutagenicity model has been developed according the US Environmental Protection Agency (EPA) Genotox protocol and assesses the probability of a compound that the query structure represents to be mutagen. The developmental toxicity potential (DTP) modules of TOPKAT assess the probability of a query structure to be a developmental toxicant in the rat. Developmental toxicity is any reversible or irreversible alteration, which affects normal growth or development or behaviour of biological organism. The rodent carcinogenicity model computes the probability of a compound that the chemical query structure represents to be carcinogen. Models that compute the probability of a compound to be an ocular irritant or a skin sensitiser were also included in the study. The rat oral LD50 module of the TOPKAT package assesses oral acute median lethal dose, LD50, in the rat. It comprises QSTR models and experimental LD50 values for approximately 4000 chemicals. All the models used were developed with structurally diverse compounds and their measured ADMET properties. In the present study ADMET and toxicity profiles for the most active compounds were studied using their 2D chemical structures as input to DS.

## Results and Discussion

### Antimicrobial activity of betulin derivatives

Primary antimicrobial screening of 51 structurally diverse betulin derivatives showed that five compounds (**4**, **5**, **23**, **35**, **38**) inhibited the growth of Gram-positive bacteria at 50 µM concentration by >70% (Tables 1, S1 and S2). Of these, 28-*O*-(*N*-acetylanthraniloyl)betulin (**5,** Fig. 1) was the most active, with >90% inhibition of both *S. aureus* and *E. faecalis*. Betulonic acid (**23**) inhibited the growth of *E. faecalis* by 74% and *S. aureus* by 51%. The active compounds were further evaluated in confirmatory dose-response experiments, and MIC_90_ value of 6.25 µM against both Gram-positive bacteria was determined for compound **5**. With all the other compounds, 90% inhibition was not achieved even at the highest test concentration 100 µM. Noteworthy, several betulin derivatives showed activity against *E. faecalis;* 25 compounds displaying moderate activity (range 50–70%) and five compounds with inhibition >70% (Fig. 1, Tables S1 and S2). All heterocyclic betulin derivatives showed some effect against *E. faecalis*, the inhibition ranging from 38 to 73% (Table S2) whereas almost all acetyl derivatives of betulin screened in our study, especially when the acetyl group was attached to 28-OH (compounds **14**, **15**, **16**, **21** and **29**; Table S1), were inactive against all microbial strains tested. None of the betulin derivatives were found to be active against Gram-negative bacteria *E. coli, P. aeruginosa* and *E. aerogenes*, with the exception of betulinic acid (**19**) and its methyl ester (**20**) which showed minor activities (38 and 39% inhibition, respectively) against *E. aerogenes.* Obviously, this might be due to the presence of outer membrane in Gram-negative bacteria, which is an efficient barrier for compound entry, whereas Gram-positive bacteria lack the outer membrane structure.

Based on the differences between the derivatives in activity against Gram-positive bacteria *S. aureus* and *E. faecalis,* the following structure-activity relationships could be observed. Starting material betulin (**1**), displayed low activity (20%) comparing to its dihydro derivative, compound **2** (62%) against *E. faecalis*. In addition, increased activity against this particular microbial strain was displayed by all betulin C28 (R_2_) monoesters (**3–9**): betulinyl 28-carboxymethoxycarvacrolate (**4**) and 28-*O*-(*N*-acetylanthraniloyl)betulin (**5**) showed significant effect (72 and 99%, respectively). Furthermore, betulinic acid (**19**) and its methyl ester (**20**) showed moderate activities (56 and 51% inhibition, respectively) against *E. faecalis*. Within the group of compounds **21–27** beneficial effect of C3 carbonyl (R_1_) was observed, with some of the derivatives showing improved activity against this Gram-positive strain: dihydrobetulonic acid (**24**; inhibition 57%), vanillinyl betulonate (**26**; 59% inhibition) and betulonic acid (**23**; 74% inhibition). Nonetheless, methyl ester of betulonic acid (**25**) was totally inactive, whereas oxime (**30** and **31**) and C28 nitrile derivatives (**32** and **33**) showed modest activity against *E. faecalis* (57, 41, 57, and 49%, respectively).

Interestingly, heterocyclic compounds (**34–41**) having acetyl groups at C3 and C28 (R_2_) with aromatic substituents at N-4 (R_1_) displayed slightly increased activity, when compared to heterocyclic compounds having bulkier acyl groups at C3 and C28 or methyl at N-4. Heterocycles with aromatic substituents at N-4, 1,3-dioxol-5-yl (**35**) and 3-NO_2_-phenyl (**38**) displayed the best activities (70 and 74% inhibition, respectively) in this particular subgroup (Table S2).

Our findings are in agreement with previous reports in which low activity has been described for betulin (**1**) against *S. pyogenes* (MIC 85 µg/mL) [11], *S. aureus* (MIC 0.25 mg/mL), *E. coli* (MIC 0.25 mg/mL) and *P. aeruginosa* (MIC 0.25 mg/mL) [32]. Similarly, betulinic acid (**19**) has been found to be inactive against *S. aureus, E. coli, B. subtilis* and *Micrococcus luteus* [19], [33], [34].

Furthermore, Krasutsky et al. have assayed 35 lupane-type triterpenes (17 allobetulin derivatives, 17 betulin derivatives and 1 lupeol derivative) using Kirk-Bauer disc diffusion method against *E. coli, S. aureus* and *B. subtilis,* as well as 9 lupane derivatives against *S. epidermidis, S. aureus* and *E. faecalis* [35]. Derivatives consisted mostly of esters (e.g. succinate, phthalate and glutarate), amides (e.g. glycine, alanine and proline), as well as phosphates and oxidation products. Betulin, allobetulin and lupeol derivatives performed very poorly, as only very few showed any activity. In addition, none of the compounds inhibited *B. subtilis* even at very high 10 mg/mL concentration. Evident beneficial effect of C3 carbonyl, observed also in our study, was noted, as lupenon-1,2-ene-2-ol and lupenone were the most active compounds and clearly more active than the parent compound lupeol (all having methyl at C28) against *E. coli* and *S. aureus* [35]. In addition, enhanced antibacterial activity of betulonic acid (**23**), as well as dihydrobetulonic acid (**24**) has been also reported against *Chlamydia pneumoniae,* as they performed in the top five compounds out of 32 betulin derivatives [16].

Other naturally occurring and closely related pentacyclic triterpenes possessing six-membered E-ring, i.e. ursolic acid (UA), oleanolic acid (OA) and morolic acid (MA) have also been studied in similar manner for their antimicrobial potential. UA and OA have been previously reported to exhibit stronger activity against *S. aureus* and *E. faecalis* than betulinic acid (UA: MIC 4–8; OA: 8–64; **19** >256 µg/mL). In addition, all three triterpenes were inactive against *E. coli* and *P. aeruginosa* [36]. Gherke et al. have reported improved activity for MA (MIC≥100 µg/mL) after introduction of C3 carbonyl group (MIC≥3.12 µg/mL), when tested against a panel of seven different bacterial species (*B. subtilis, S. aureus, S. pyogenes, Staphylococcus saprophyticus, E. coli, P. aeruginosa* and *Shigella sonnei*) [37]. The beneficial effect of C3 carbonyl group could be partially explained by the crystal structure of these triterpenes. Betulonic acid (**23**) in DMSO–water (9∶1, v/v) solvatomorphs reveal that the ring-A with a C3 carbonyl adopts a flattened twisted-boat conformation, which differs from betulin derivatives having a C3 hydroxyl group at this position. It has been speculated, that the orientation of the rings can influence hydrogen bonding and the other interactions of betulonic acid (**23**) [38].

To our knowledge, UA or OA having a C3 carbonyl and C28 carboxyl groups have not been assayed against any bacterial species, even though both have been used as a starting material in studies of related derivatives against *Mycobacterium tuberculosis* [39], *S. aureus* and *Klebsiella pneumoniae* [40]. It would be highly interesting to investigate their antibacterial properties to further evaluate the hypothesis of favouring effect of both C3 carbonyl and C28 carboxyl groups in pentacyclic triterpenes.

In this study, the set of 51 betulin derivatives was also tested against *C. albicans.* Most of them, including starting material betulin (**1**), as well as betulinic acid (**19**), were inactive but some beneficial effect could be observed with C28 esters, e.g. betulin esters 28-*O*-chrysanthemoylbetulin (**3**), betulinyl 28-carboxymethoxycarvacrolate (**4**) and 28-*O*-nicotinoylbetulin (**6**) showed minor activities (31–37% inhibition; Table S1). Moreover, betulinic or betulonic acid derived compounds, i.e. betulinic acid methyl ester (**20**), dihydrobetulonic acid (**24**) and L-aspartyl amide of betulonic acid (**27**), displayed modest inhibitory activity against this fungal strain (inhibitory effects 34–55%). Noteworthy, all heterocyclic betulin derivatives (**34–51**; Table S2) were inactive. Comparable results, presenting low antifungal activity against *C. albicans,* have been previously reported for betulin (**1**) and its derivatives (i.e. allobetulin and lupeol derivatives) [35] (MIC 250 µg/mL) [12] and (MIC 180 µg/mL) [41].

Despite the numerous reports on the antimicrobial activity displayed by pentacyclic triterpenoids of lupane skeleton i.e. betulin and its derivatives, their mechanism of action is still not fully understood. Sathya Bama et al. have recently reported significant antimicrobial activity of triterpenoid extract from the leaves of *Tridax procumbens* L., with lup-20(29)-en-3*β*-ol as the most active component [42]. Response mechanism to pentacyclic triterpenoids possessing lupane skeleton was described to involve changes in membrane permeability arising from the leakage of reducing sugars and proteins, as well as reduction of respiratory chain dehydrogenase enzyme’s activity. Noteworthy, among four tested microbial strains (*S. aureus*, *E. coli*, *Proteus mirabilis* and *Vibrio cholerae*), *S. aureus* was the most susceptible to the treatment with the active compound [42]. Reported approach presents the great potential of pentacyclic triterpenoids with an isopropenyl moiety and simultaneously suggests the possible mode of action for compounds sharing lupane skeleton, including the derivative **5**.

### Evaluation of antimicrobial activity in a host-pathogen co-culture assay

**Figure 2:**
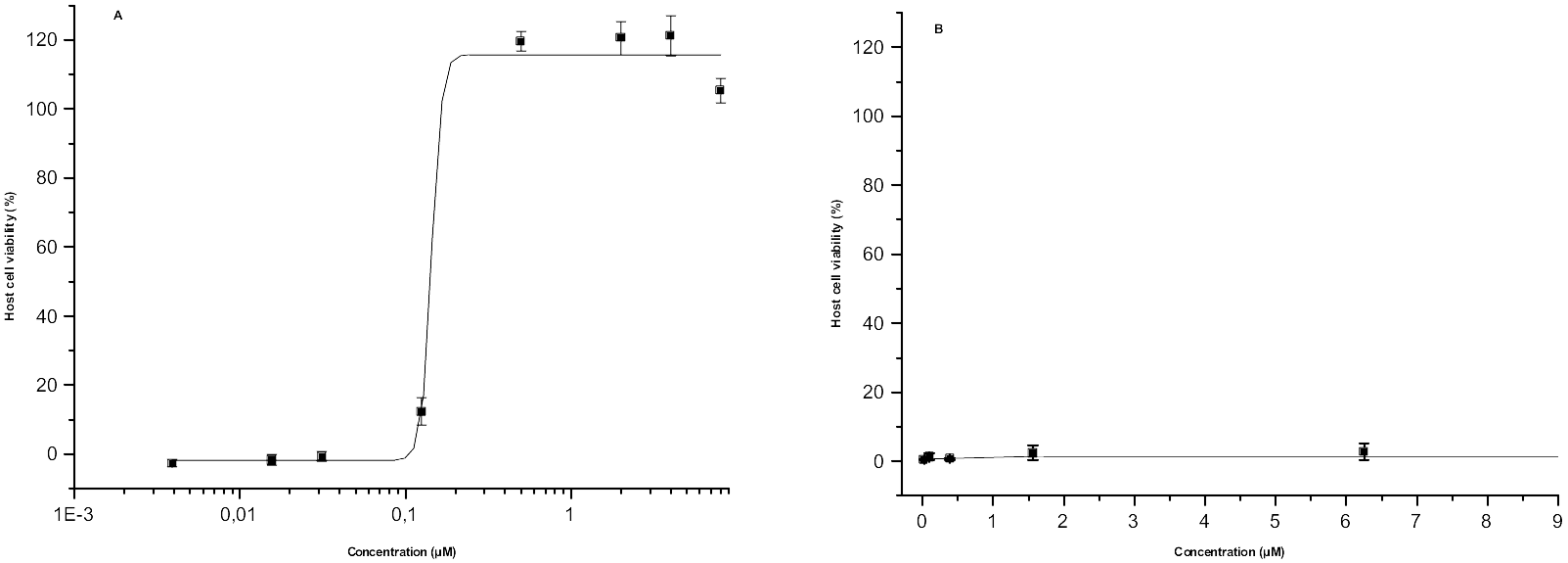
Antimicrobial activity in host-pathogen co-culture assay. The assay was based on *S. aureus* infected HL cell line. Dose-response results for a) ciprofloxacin) and b) 28-*O*-(*N*-acetylanthraniloyl)betulin (**5**).

For studying simultaneously the efficacy and tolerability of antibacterial compounds, an assay based on co-culturing of a secondary human cell line (HL, derived originally from human lung) and a pathogen was used. This system mimics natural bacterial infection, and gives thus more profound information on the antibacterial potential of test compounds. In the assay, host cells are incubated in the presence of the test sample and are infected with bacteria. After 3 days of incubation, the host cell survival is determined using fluorescein diacetate to measure the viability of the host cells. As outputs, the assay provides both half-maximal inhibitory concentrations (IC_50_) and half-maximal cytotoxic concentrations (CC_50_) for the test compound. During assay validation, both the host cell and the pathogen culturing was optimised (number of cells used, inoculum size, incubation time; data not shown). Fig. 2a presents results obtained with the reference compound, ciprofloxacin.

After assay validation, the host-pathogen co-culture assay was used to evaluate the antibacterial activity of the two most potent betulin derivatives, 28-*O*-(*N*-acetylanthraniloyl)betulin (**5**) and betulonic acid (**23**). Compounds were assayed at seven different concentrations in the range of 0.024–100 µM. Surprisingly, no activity was detected with compound **5**, and compound **23** showed only low activity (27%) at the highest concentration. Data obtained for compound **5** is displayed in Fig. 2b as an example.

### Effect of BSA on antimicrobial activity

As described above, the antimicrobial activities of betulin derivatives seen in the microdilution assay were not detectable in the host-pathogen co-culture assay. Since the assay was performed in cell culture media containing 7.5% of fetal bovine serum, this was suspected to be the reason. Serum is rich in proteins, the most abundant of these being albumin. First, we performed the normal antibacterial assay by replacing the Mueller Hinton II broth with the cell culture media. This confirmed our hypothesis: no activity was detected. In order to clearly demonstrate that albumin was causing the loss of activity, we performed the microdilution assay by adding BSA at different concentrations together with the active compounds at MIC_90_. The activity of compound **5** was clearly affected by the added BSA: at 24 nM concentration the activity was reduced by 50% and at concentration 390 nM the activity was completely lost. As a comparison, the concentration of albumin in human serum is typically 36–48 g/L which corresponds to 5.42–7.22 mM concentration. Albumin is capable of interacting with many organic and inorganic molecules and functions as a key controller of intercellular fluxes [43]–[52]. Majority of therapeutic compounds are reversibly bound to plasma proteins, but the degree and nature of the binding differs. The degree of binding between a drug and serum proteins governs its therapeutic potential and pharmacokinetic performance, and albumin binding is usually considered as a disadvantage for a drug candidate. Betulinic acid (**19**) has been previously reported to bind to human serum albumin [53], which supports our findings for the derivative **5**. Investigations on, for example, the binding affinity of compound **5** to the BSA molecule, would further elucidate the significance of this feature.

### Effects on mammalian cells and *in silico* predictions of ADMET properties

**Figure 3:**
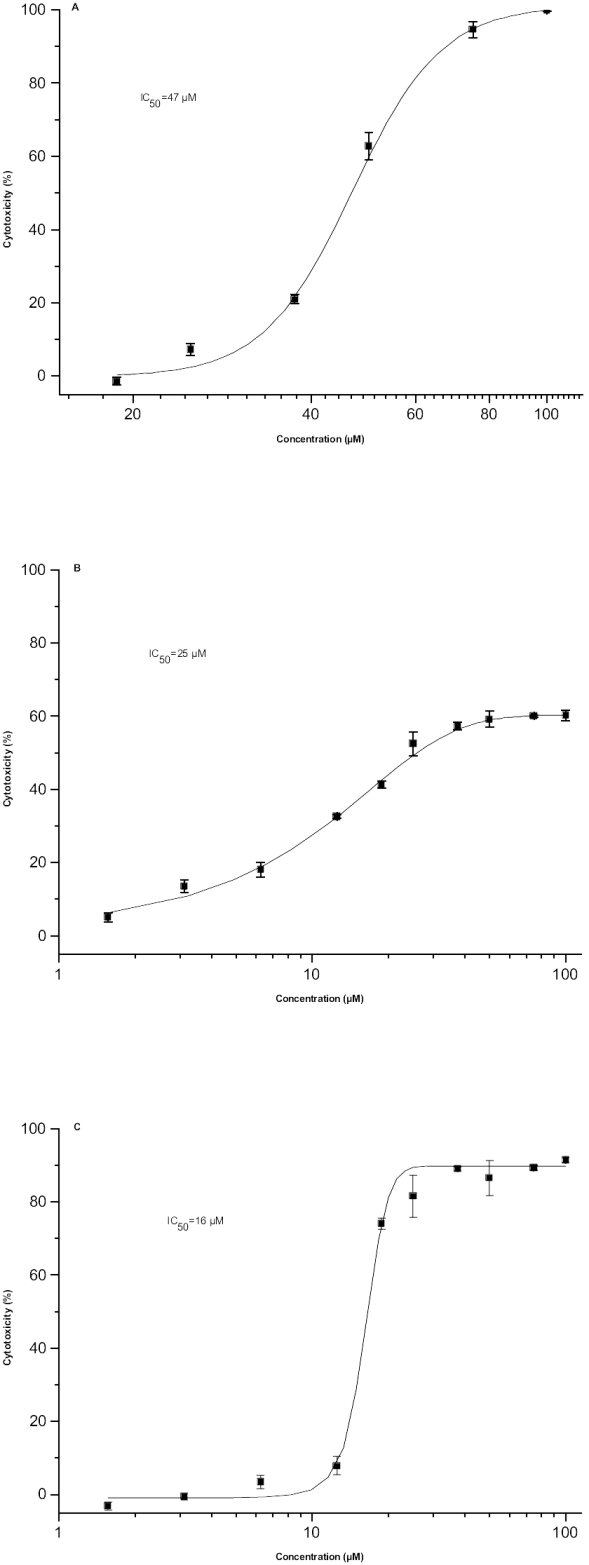
Cytotoxic effect of selected betulins on mammalian cells (Huh-7) after 24 h exposure at different concentrations. A) betulinic aldehyde (**18**), B) betulin-28-oxime (**31**) and C) hetero cycloadduct (**43**).

In order to further characterise the potential of betulin derivatives as antibacterial agents, selectivity evaluation between prokaryotic and eukaryotic cells (cytotoxicity assays on hepatocyte Huh-7 cell line) and *in silico* predictions of ADMET properties were carried out. Measuring the intracellular ATP content is a very sensitive and common method for evaluating cell viability after compounds exposure. The cells were exposed to the compounds for 24 h, which necessitated the use of FBS-containing cell culture media in the experiment, but the serum concentration was reduced from 10 to 5% in order to decrease the possible interference of serum albumin. In the primary testing at 50 µM concentration, following compounds displayed the highest cytotoxicity towards Huh-7 cells: hetero cycloadduct (**43**; cytotoxicity 85%), betulinic aldehyde (**18**; cytotoxicity 61%) and betulin-28-oxime (**31**; cytotoxicity 61%) (Table 1). Cytotoxicities of 28-*O*-(*N*-acetylanthraniloyl)betulin (**5**) and betulonic acid (**23**) were 30 and 18%, respectively. IC_50_ values were determined for the most cytotoxic derivatives (**18**, **31** and **43**) and for the most antibacterially active derivative **5**. Polymyxin B sulphate was used as a positive control (IC_50_ of 9361 IU/mL). As could be expected from the primary results presented in Table 1, three compounds exhibited clear cytotoxicity: IC_50_ values of 47, 25 and 16 µM were measured for compounds **18**, **31** and **43** (Fig. 3), respectively. In addition, the IC_50_ value of 56 µM for compound **5** was determined. Conclusions on the selectivity of compound **5** towards bacteria cannot, however, be made based on this finding. The presence of 5% serum in the cytotoxicity assay and thus albumin binding may have influenced the detected cytotoxicity. Furthermore, the ATP assay showed that at higher concentrations (100 µM), compounds **5**, **18** and **43** completely destroyed the cell monolayer, whereas compound **31** showed only 60% of inhibition.

The cytotoxic effect towards human Huh-7 hepatocyte cells observed for betulinic aldehyde (**18**), as well as hetero cycloadduct (**43**) is in agreement with previous reports [20], [21]. Betulin-derived compounds have been reported to be cytotoxic towards cancer cell lines of different histogenetic origin, including drug resistant tumours [54], [55]. For instance, betulinic acid (**19**) have been shown to display strongly selective effect against human melanoma cell lines (A375, 518A2, MES20 and MES21) [56], [57] by inducing apoptosis via the activation of caspases and having direct effects on mitochondria [55], [58]–[60]. However, its cytotoxicity against hepatocytes observed in our study was quite low (30% inhibition), which is in agreement with the previous reports [20] and confirms that its activity is strongly dependent on the type of cancer cell line [60]. Noteworthy, in contrast to betulinic acid (**19**), betulin (**1**) has no significant cytotoxic effect against various tumour cell lines [61], which was also observed in our study (compound **1**, cytotoxicity 14%). In addition, it was previously reported that oxidation of betulin to betulinic aldehyde (**18**) results in significantly increased cytotoxicity against cancer cell lines [55]. Moreover, betulin 3,28-dioxime was cytotoxic against human oral epidermoid carcinoma (KB), whereas betulinic acid 3-oxime was inactive, indicating the importance of the suitable substitution pattern at C28 for the activity [61].

Compound instability in experimental conditions may lead to loss of activity, or to production of metabolites, which can be more active than the original compound. Betulin **1** is a stable compound, as we have not noticed any degradation of samples even if stored at ambient temperature under air atmosphere for several years. Moreover, the most unstable chemical bond within betulin derivatives (Fig. 1), the ester bond, is stable under identical incubation conditions as demonstrated by Zenger et al. [62] with succinic acid-bridged tacrine–silibinin co-drug. In addition, other chemical bonds such as amide and ether were shown to be stable as well. Thus, instability issues cannot be seen as a major concern regarding the betulin derivatives of this study.

Prediction of ADMET properties *in silico* can be a good tool to further evaluate compounds’ potential and may be used to eliminate compounds possessing unfavourable characteristics at early phase in the drug discovery process. This will help later to optimise and prioritise the most promising compounds for further hit to lead optimisation. For this reason, we carried out computational evaluation of ADMET properties [human intestinal absorption (HIA), aqueous solubility, blood brain barrier, cytochrome P450 2D6 inhibition, hepatotoxicity and plasma protein binding] and prediction of several toxicity end-points for the most active compounds. According to the predicted values, the derivatives differ in their predicted absorption and solubility properties, but show similar tendency for cytochrome P450 2D6 inhibition, hepatotoxicity, plasma protein binding and toxicity end-points (Tables S3 and S4). Toxicity potential prediction also shows some difference between these compounds. The ADMET plot (Fig. S1) using calculated PSA (polar surface area) versus AlogP98 properties contains two sets of ellipses, which represent the prediction confidence space (90% and 99%) for the BBB and HIA models, respectively. In ADMET plot, for the BBB property, six of the nine compounds are outside the 99% ellipse (undefined, Table S3), only two compounds (compound **1** and **18**) are inside the 99% ellipse (high, Table S3). Hence, most of the compounds are most likely not able to penetrate the BBB and the possibility of CNS side effects are absent. Only the two compounds with high BBB value represent a possible risk, as they are predicted to easily cross the BBB. The analysis of the plot for the HIA property reveals that four compounds (compounds **1**, **18**, **23**, **31**) fall inside the 99% confidence ellipse, whereas the rest of the compounds are outside. This means that these four compounds possess good HIA. All compounds are predicted to have low solubility, to be non-inhibitor of CYP2D6 and without hepatotoxicity. Eight of the nine compounds (compounds **1** to **38**) are predicted to bind highly to carrier proteins in the blood, which we also observed in the experiments in the presence of BSA.

Some of the predicted toxicity end-points are resumed in Table S4. TOPKAT suggests that most of the compounds are aerobically biodegradable, all are non-mutagen and devoid of any carcinogenicity, ocular irritancy and skin irritancy. Six of the nine compounds present developmental toxicity potential (compounds **1** to **31**). However, TOPKAT models tend to overestimate the toxicity of compounds by assigning undetermined value as toxic. The predicted rat oral LD50 values of all the compounds range from 0.406 g/kg to 5.130 g/kg. These high LD50 values suggest that these compounds are lethal only at very high dose. For all models, an analysis was performed on query compounds before the prediction, to ensure that they are within the optimum prediction space in which the model is applicable. All the compounds are within the optimum prediction space of models. This analysis gives an indication of the potential effect of these compounds on certain toxicity end-points. With this information, selected compounds can be further explored for structural feature identification and optimization to improve their activity and toxicity potential. In summary, according to predicted ADMET properties (Table S3), compounds **23** and **31** can be favoured in the view of their good predicted properties.

## Conclusions

In this study, primary screening and a set of follow-up studies have been used for evaluating the potential antimicrobial action of betulin derived compounds. Primary antimicrobial screening showed that 28-*O*-(*N*-acetylanthraniloyl)betulin (**5**) was the most active showing MIC_90_ of 6.25 µM against two Gram-positive bacteria, *E. faecalis* and *S. aureus*. Nevertheless, its activity was affected by albumin binding, which was demonstrated by loss of activity in the host-pathogen co-culture assay as well as in the antibacterial assay in the presence of increased concentration of albumin. Follow-up cytotoxicity studies, based on ATP measurement of human hepatocyte cell culture after 24 h exposure to the compounds, showed that three compounds, betulinic aldehyde (**18**), betulin-28-oxime (**31**) and hetero cycloadduct (**43**) displayed cytotoxicity towards hepatocytes. This study demonstrated that introduced modifications to the parent betulin structure can result in varying level of antibacterial activity and cytotoxicity, and the right choice of substitution is crucial for the improvement of their pharmacological properties.

Most importantly, the results demonstrate the significance of taking a multidimensional approach when studying biological activities of new compounds. Excluding the follow-up studies carried out on mammalian cells and in *in silico* would have given a very limited view on the real potential of the hits identified in the antimicrobial screening.

On the other hand, previous reports on combined antibiotic therapy of triterpenoids, including betulin derivatives (i.e. betulinic acid and betulinic aldehyde), with commonly available antibiotics could be also seen as an alternative way for applying compounds that show relatively modest antimicrobial effects [63]. Pentacyclic triterpenoids in combination with reference antibiotics could target different sites in bacteria and in such a way generate either an additive or synergistic effect, as well as contribute to the delay in the appearance of antimicrobial resistance.

## Supporting Information

Figure S1 **ADMET plot for both blood brain barrier (BBB) and human intestinal absorption (HIA).**
(DOCX)

Table S1 **Primary screening results for compounds 1–33 at 50 µM concentration.**
(DOCX)

Table S2 **Primary screening results for compounds 34–51 at 50 µM concentration.**
(DOCX)

Table S3 ***In silico*** **predicted ADMET properties for the most active betulin derivatives.**
(DOCX)

Table S4 ***In silico*** **predicted toxicity probabilities for the most active betulin derivatives by TOPKAT.**
(DOCX)
