# Supplementary figures and images for: Screening and Characterisation of Antimicrobial Properties of Semisynthetic Betulin Derivatives
Source: PLoS One. 2014 Jul 17;9(7):e102696. doi: 10.1371/journal.pone.0102696 (PMC4102551; doi:10.1371/journal.pone.0102696)

**Figure S1.** ADMET plot for both blood brain barrier (BBB) and human intestinal absorption (HIA).


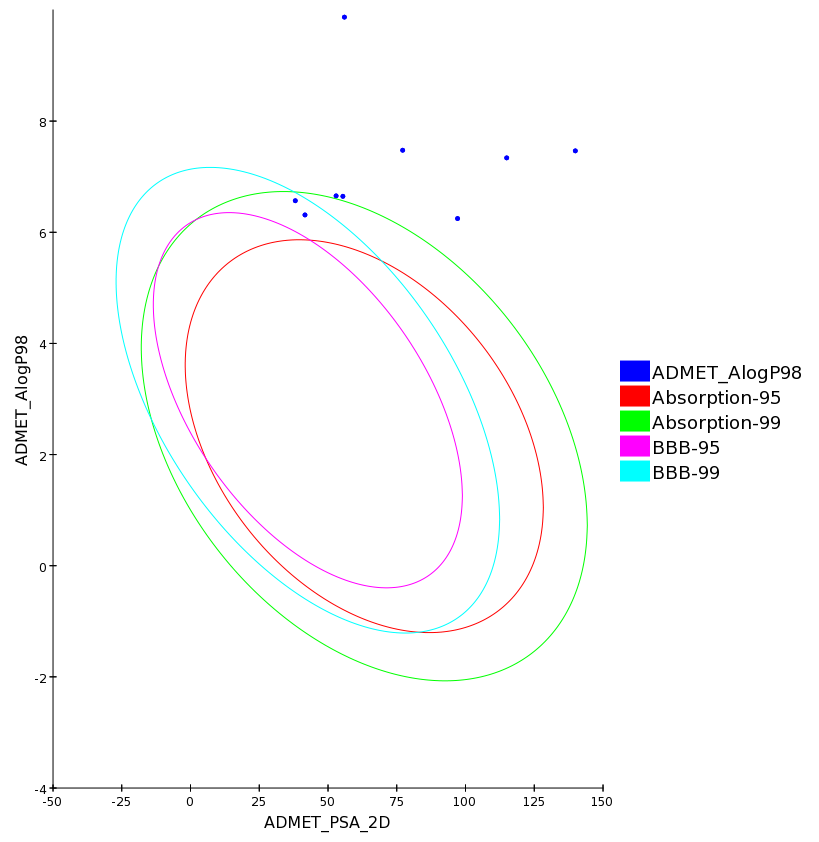

Supplement: Figure S1 — ADMET plot for both blood brain barrier (BBB) and human intestinal absorption (HIA). (DOCX) [file pone.0102696.s001.docx]
