# Supplementary material for: Screening and Characterisation of Antimicrobial Properties of Semisynthetic Betulin Derivatives
Source: PLoS One. 2014 Jul 17;9(7):e102696. doi: 10.1371/journal.pone.0102696 (PMC4102551; doi:10.1371/journal.pone.0102696)
Supplement: Table S1 — Primary screening results for compounds 1–33 at 50 µM concentration. (DOCX) [file pone.0102696.s002.docx]

**Table S1.** Primary screening results for compounds **1**-**33** at 50 µM concentration (colour description: yellow - effect 30-50%; green - effect 50-70%; red – effect >70%).

| ***Compound*** | ***R_1_*** | ***R_2_*** | ***R_3_*** | ***Inhibitory effect (%)*** | | | | | | ***Cytotoxicity (%)*** |
| --- | --- | --- | --- | --- | --- | --- | --- | --- | --- | --- |
|  |  |  |  | ***E. aerogenes ATCC 13048*** | ***E. coli***  ***ATCC 25922*** | ***E. faecalis***  ***ATCC 29212*** | ***P. aeruginosa ATCC 27853*** | ***S. aureus***  ***ATCC 25923*** | ***C. albicans***  ***ATCC 90028*** | ***Huh-7 cells*** |
| **1** | OH | CH_2_OH | CH_3_-C=CH_2_ | 5.7±2.1 | 1.3±5.1 | 19.7±6.2 | -1.2±2.5 | -6.7±6.4 | -27.5±15.0 | 13.7±2.7 |
| **2** | OH | CH_2_OH | (CH_3_)_2_CH- | 13.4±4.2 | 1.3±3.5 | 62.2±2.5 | 2.0±1.0 | 7.8±1.4 | -3.7±9.9 | 28.0±7.5 |
| **3** | OH |  | CH_3_-C=CH_2_ | 10.6±0.5 | 0.3±1.7 | 46.9±6.0 | 4.4±1.4 | -23.2±11 | 35.4±3.8 | -5.8±2.5 |
| **4** | OH |  | CH_3_-C=CH_2_ | 9.9±3.3 | -1.2±1.2 | 72.3±4.0 | 3.0±3.0 | -17.2±0.7 | 31.4±9.1 | -4.6±3.6 |
| **5** | OH |  | CH_3_-C=CH_2_ | 25.2±2.0 | 0.5±3.1 | 98.5±0.2 | 12.3±2.3 | 100.2±0.1 | 10.0±5.2 | 30.0±3.0 |
| **6** | OH |  | CH_3_-C=CH_2_ | 7.5±1.3 | -0.6±6.8 | 60.1±4.0 | 2.2±2.2 | -17.7±2.0 | 36.7±7.6 | -0.4±3.4 |
| **7** | OH |  | CH_3_-C=CH_2_ | 12.6±0.7 | -4.8±4.7 | 43.5±12.0 | 2.6±5.4 | -30.9±9.4 | -40.3±25.1 | -10.4±3.3 |
| **8** | OH |  | CH_3_-C=CH_2_ | 12.8±3.0 | 4.8±4.2 | 56.6±1.8 | 3.4±1.4 | 22.9±3.1 | 24.0±14.3 | 22.4±8.5 |
| **9** | OH | CH_2_OAc | CH_3_-C=CH_2_ | 9.1±2.1 | -0.2±7.1 | 50.9±2.1 | -0.6±3.3 | 5.1±6.9 | 3.0±10.1 | 5.1±1.0 |
| **10** | OH |  | CH_3_-C=CH_2_ | 8.1±2.9 | -4.0±0.8 | 61.0±6.1 | 4.1±1.7 | -9.2±1.5 | -11.8±7.8 | -4.6±3.2 |
| **11** | OAc |  | CH_3_-C=CH_2_ | 11.2±0.5 | -7.0±3.5 | 52.0±0.7 | 2.6±0.4 | -31.2±2.4 | -9.2±23.1 | -6.6±3.3 |
| **12** | OAc | CH_2_OH | CH_3_-C=CH_2_ | 14.5±1.3 | 1.0±5.0 | 54.7±7.9 | 1.8±1.2 | -16.3±7.4 | -18.0±27.4 | 3.8±14.1 |
| **13** | OAc | CH_2_OMs | CH_3_-C=CH_2_ | 15.0±1.9 | -0.4±3.4 | 61.2±1.5 | 6.5±0.9 | -16.5±3.0 | -14.8±8.5 | 0.2±3.8 |
| **14** | OAc | CH_2_OAc | CH_3_-C=CH_2_ | 8.1±9.0 | -1.8±2.1 | 22.8±17.5 | 3.9±0.6 | 13.8±3.2 | -1.1±18.3 | -11.1±3.8 |
| **15^a^** | OAc | CH_2_OAc | (CH_3_)_2_CH- | 13.3±3.3 | -3.4±2.5 | 11.7±4.1 | 3.7±1.6 | 14.7±8.9 | 0.5±15.5 | -9.0±2.3 |
| **16^a^** | OAc | CH_2_OAc | (CH_3_)_2_CH- | 7.6±9.0 | 3.0±0.9 | 14.7±4.5 | 3.0±0.7 | 24.2±2.7 | 16.0±13.3 | -6.4±4.7 |
| **17** |  |  | CH_3_-C=CH_2_ | 7.4±1.9 | -0.6±3.7 | 24.3±5.6 | 3.7±1.0 | 1.7±0.5 | 21.7±9.6 | -2.6±3.3 |
| **18** | OH | CHO | CH_3_-C=CH_2_ | 13.5±2.6 | 2.7±2.4 | 39.6±8.0 | 3.9±0.6 | -35.5±3.2 | 6.9±8.1 | 61.3±9.5 |
| **19** | OH | CO_2_H | CH_3_-C=CH_2_ | 38.2±3.0 | 20.2±6.1 | 55.9±5.0 | 6.7±1.4 | 22.8±5.4 | 21.6±2.6 | 31.6±15.7 |
| **20** | OH | CO_2_Me | CH_3_-C=CH_2_ | 38.9±1.5 | 13.5±8.9 | 51.1±0.6 | 8.6±2.5 | 5.3±4.2 | 34.3±5.6 | 33.7±7.0 |
| **21** | O= | CH_2_OAc | CH_3_-C=CH_2_ | 12.6±6.2 | 2.6±1.8 | 5.7±13.4 | 4.7±2.0 | 0.7±3.4 | -13.9±24.7 | -3.9±1.1 |
| **22** | O= | CHO | CH_3_-C=CH_2_ | 6.0±4.6 | -1.2±0.5 | 16.3±19.0 | 4.5±1.6 | 0.1±1.5 | -18.7±15.1 | 4.1±17.7 |
| **23** | O= | CO_2_H | CH_3_-C=CH_2_ | 21.8±4.1 | 9.1±8.9 | 74.3±0.8 | 9.5±2.5 | 51.1±7.3 | 25.2±11.0 | 17.8±12.6 |
| **24** | O= | CO_2_H | (CH_3_)_2_CH- | 16.8±5.4 | 6.2±4.5 | 56.6±2.0 | 4.9±0.5 | 15.1±3.7 | 54.9±8.3 | 13.0±10.3 |
| **25** | O= | CO_2_Me | CH_3_-C=CH_2_ | 11.4±3.9 | -3.3±1.4 | -3.4±5.9 | 2.3±0.3 | 2.5±1.3 | -30.0±17.3 | 23.0±4.0 |
| **26** | O= |  | CH_3_-C=CH_2_ | 7.5±0.6 | 4.1±0.4 | 58.7±0.1 | 6.6±1.0 | 7.3±5.8 | -65.9±15.0 | -10.1±2.2 |
| **27** | O= |  | CH_3_-C=CH_2_ | 9.3±1.0 | 3.1±4.6 | 38.2±15.5 | -1.2±0.4 | 12.7±3.3 | 44.2±3.8 | 10.9±1.3 |
| **28^a^** | - | CH_2_OH | CH_3_-C=CH_2_ | 12.2±1.0 | -4.7±0.6 | 49.2±4.6 | -0.6±3.1 | 7.8±1.7 | -29.5±18.6 | -4.5±0.7 |
| **29^a^** | - | CH_2_OAc | CH_3_-C=CH_2_ | 11.2±7.1 | 2.8±3.0 | 20.9±15.7 | 5.7±1.6 | 11.4±4.3 | -10.0±27.1 | -11.3±3.6 |
| **30** | =NOH | CH=NOH | CH_3_-C=CH_2_ | 18.5±3.4 | 7.9±6.1 | 56.9±2.6 | 1.4±1.1 | 5.1±6.0 | 13.9±8.8 | 42.4±5.4 |
| **31** | OH | CH=NOH | CH_3_-C=CH_2_ | 14.6±0.9 | 1.9±4.0 | 41.0±3.4 | 5.4±2.2 | 6.4±3.4 | -73.5±43.9 | 61.5±2.0 |
| **32** | =NOAc | CN | CH_3_-C=CH_2_ | 12.0±3.0 | 5.5±1.5 | 56.6±7.0 | 5.7±1.7 | 5.5±1.9 | -43.8±12.6 | -7.2±3.0 |
| **33** | OAc | CN | CH_3_-C=CH_2_ | 11.4±4.2 | -4.6±3.3 | 49.2±2.2 | 3.6±3.9 | 20.5±52.1 | -51.2±39.1 | 21.6±17.0 |

^a^See chemical structures above.
