# Supplementary material for: Screening and Characterisation of Antimicrobial Properties of Semisynthetic Betulin Derivatives
Source: PLoS One. 2014 Jul 17;9(7):e102696. doi: 10.1371/journal.pone.0102696 (PMC4102551; doi:10.1371/journal.pone.0102696)
Supplement: Table S2 — Primary screening results for compounds 34–51 at 50 µM concentration. (DOCX) [file pone.0102696.s003.docx]

**Table S2.** Primary screening results for compounds **34**-**51** at 50 µM concentration (colour description: yellow - effect 30-50%; green - effect 50-70%; red – effect >70%).

| ***Compound*** | ***R_1_*** | ***R_2_*** | ***Inhibitory effect (%)*** | | | | | | ***Cytotoxicity (%)*** |
| --- | --- | --- | --- | --- | --- | --- | --- | --- | --- |
|  |  |  | ***E. aerogenes***  ***ATCC 13048*** | ***E. coli***  ***ATCC 25922*** | ***E. faecalis***  ***ATCC 29212*** | ***P. aeruginosa***  ***ATCC 27853*** | ***S. aureus***  ***ATCC 25923*** | ***C. albicans***  ***ATCC 90028*** | ***Huh-7 cells*** |
| **34** | 3-MeO-Ph | Ac | 13.8±4.3 | 1.7±4.2 | 68.4±3.0 | 0.6±2.5 | 3.1±2.0 | -1.1±3.5 | 8.5±2.0 |
| **35** | 1,3-dioxol-5-yl | Ac | 14.2±1.6 | 3.4±3.1 | 70.0±0.9 | 5.5±1.0 | 5.9±1.2 | 4.4±12.9 | -0.1±3.6 |
| **36** | indan-5-yl | Ac | 14.5±1.3 | 2.6±2.1 | 62.0±1.9 | 5.8±0.7 | 6.1±1.7 | 10.7±4.8 | 7.9±2.2 |
| **37** | 4-F-Ph | Ac | 17.7±2.2 | 5.0±8.8 | 48.2±7.7 | 4.6±0.7 | 6.5±0.9 | 11.7±9.0 | 0.8±3.4 |
| **38** | 3-NO_2_-Ph | Ac | 19.3±3.0 | 8.8±2.6 | 73.9±1.4 | 7.6±1.4 | 6.7±1.5 | -13.8±13.3 | 8.3±3.9 |
| **39** | 3-Cl-Ph | Ac | 19.0±1.9 | 4.4±2.7 | 69.1±4.9 | 2.6±1.1 | 5.7±0.8 | 4.8±13.6 | 1.5±2.7 |
| **40** | Bn | Ac | 15.1±3.4 | -0.8±2.3 | 66.0±1.5 | 1.7±0.6 | 4.1±1.1 | 4.4±10.5 | 2.3±2.2 |
| **41** | Ph | Ac | 18.6±5.6 | 7.1±1.0 | 52.4±7.2 | 1.3±0.4 | 12.7±1.6 | 15.0±14.3 | 1.3±4.4 |
| **42** | *n*-Bu | Ac | 10.6±3.5 | 1.7±5.2 | 67.5±3.7 | 1.0±0.4 | 1.5±1.0 | -12.0±11.8 | -8.3±1.3 |
| **43** | Et | Ac | 18.5±2.2 | -3.7±4.3 | 57.1±4.4 | 1.7±1.7 | 2.9±0.7 | -9.7±14.1 | 86.0±2.0 |
| **44** | Ph | H | 11.7±1.1 | -1.0±0.3 | 57.1±7.1 | 3.3±1.1 | 5.7±2.2 | -25.4±8.0 | 2.8±5.3 |
| **45** | Me | COEt | 10.8±6.9 | -4.3±7.0 | 41.6±2.8 | 3.7±1.2 | 0.7±3.8 | -30.7±19.4 | 11.1±5.1 |
| **46** | Me | COPr | 7.4±5.9 | -2.1±6.4 | 44.8±2.8 | 5.2±0.7 | 2.5±3.1 | -56.1±14.6 | 6.4±2.9 |
| **47** | Me | CO*i*-Pr | 9.6±1.7 | -3.8±4.2 | 47.8±1.3 | 1.4±0.8 | 2.9±1.0 | 12.0±12.7 | -4.9±2.2 |
| **48** | Me | COcHex | 12.1±2.2 | -5.5±1.9 | 56.3±1.8 | 7.1±1.7 | 5.0±4.2 | -39.6±25.9 | -10.8±1.9 |
| **49** | Me | COPh | 14.2±1.5 | 0.0±6.0 | 64.6±0.8 | 5.3±1.9 | 15.1±1.0 | -48.0±24.5 | -10.6±4.1 |
| **50** | Ph | COPh | 9.6±4.2 | -2.8±5.2 | 38.8±5.8 | 3.2±0.3 | 10.1±1.0 | -35.2±31.4 | -6.2±3.7 |
| **51** | *t*-Bu | COcHex | 10.8±2.5 | -3.2±1.0 | 62.3±0.3 | 5.9±0.3 | 7.5±3.8 | -96.4±22.3 | -10.0±2.6 |
