# Supplementary material for: Screening and Characterisation of Antimicrobial Properties of Semisynthetic Betulin Derivatives
Source: PLoS One. 2014 Jul 17;9(7):e102696. doi: 10.1371/journal.pone.0102696 (PMC4102551; doi:10.1371/journal.pone.0102696)
Supplement: Table S3 — In silico predicted ADMET properties for the most active betulin derivatives. (DOCX) [file pone.0102696.s004.docx]

**Table S3.** *In silico* predicted ADMET properties for the most active betulin derivatives.

| ***Compound*** | ***Human Intestinal Absorption^a^*** | ***Aqueous Solubility^b^*** | ***Blood Brain Barrier^c^*** | ***Cytochrome P450 2D6 (CYP2D6)*** | ***Hepatotoxicity*** | ***Plasma Protein Binding (PPB)*** |
| --- | --- | --- | --- | --- | --- | --- |
| **1** | Moderate | Low | High | Non-inhibitor | No hepatotoxicity | Yes |
| **4** | Very poor | Very low, but possible | Undefined | Non-inhibitor | No hepatotoxicity | Yes |
| **5** | Very poor | Low | Undefined | Non-inhibitor | No hepatotoxicity | Yes |
| **18** | Moderate | Very low, but possible | High | Non-inhibitor | No hepatotoxicity | Yes |
| **23** | Poor | Very low, but possible | Undefined | Non-inhibitor | No hepatotoxicity | Yes |
| **31** | Poor | Low | Undefined | Non-inhibitor | No hepatotoxicity | Yes |
| **35** | Very poor | Very low, but possible | Undefined | Non-inhibitor | No hepatotoxicity | Yes |
| **38** | Very poor | Very low, but possible | Undefined | Non-inhibitor | No hepatotoxicity | Yes |
| **43** | Poor | Low | Undefined | Non-inhibitor | No hepatotoxicity | No |

*^a^*Definitions for terms used:

Good = Absorption < 6.1261 (inside 95%)

Moderate = 6.1261 ≤ Absorption < 9.6026 (inside 99%)

Poor = 9.6026 < Absorption (outside 99%)

Very poor = PSA ≥ 150.0 or AlogP98 ≤ -2.0 or AlogP98 ≥ 7.0

Note: Absorption is the Mahalanobis distance for the compound in the PSA, AlogP98 plane. It is referenced from the centre of the region of chemical space defined by well-absorbed compounds

*^b^*Definitions for terms used:

Extremely low = log(Sw) < -8.0

Very low, but possible = -8.0 < log(Sw) < -6.0

Low = -6.0 < log(Sw) < -4.0

Good = -4.0 < log(Sw) < -2.0

Optimal = -2.0 < log(Sw) 0.0

No, too soluble = 0.0 < log(Sw)

*^c^*Definitions for terms used:

Very = High Brain-Blood ratio greater than 5:1

High = Brain-Blood ratio between 1:1 and 5:1

Medium = Brain-Blood ratio between 0.3:1 and 1:1

Low = Brain-Blood ratio less than 0.3:1

Undefined = Outside 99% confidence ellipse
