# Supplementary material for: Screening and Characterisation of Antimicrobial Properties of Semisynthetic Betulin Derivatives
Source: PLoS One. 2014 Jul 17;9(7):e102696. doi: 10.1371/journal.pone.0102696 (PMC4102551; doi:10.1371/journal.pone.0102696)
Supplement: Table S4 — In silico predicted toxicity probabilities for the most active betulin derivatives by TOPKAT. (DOCX) [file pone.0102696.s005.docx]

**Table S4.** *In silico* predicted toxicity probabilities***^a^*** for the most active betulin derivatives by TOPKAT.

| ***Compound*** | ***Aerobic Biodegradability*** | ***Ames Mutagenicity*** | ***Developmental Toxicity Potential*** | ***NTP^b^ Rodent Carcinogenicity*** | ***Ocular Irritancy*** | ***Skin Irritancy*** | ***Rat Oral LD50***  (g/kg body weight) |
| --- | --- | --- | --- | --- | --- | --- | --- |
| **1** | Yes | No | Yes | No | No | No | 3.267 |
| **4** | Yes | No | Yes | No | No | No | 0.406 |
| **5** | Yes | No | Yes | No | No | No | 5.130 |
| **18** | Yes | No | Yes | No | No | No | 2.160 |
| **23** | Yes | No | Yes | No | No | No | 1.940 |
| **31** | Yes | No | Yes | No | No | No | 0.708 |
| **35** | Yes | No | No | No | No | No | 2.256 |
| **38** | No | No | No | No | No | No | 2.519 |
| **43** | Yes | No | No | No | No | No | 2.264 |

*^a^*Yes = probable, No = improbable; *^b^*NTP = the U.S. National Toxicology Program
